# Deep Encoder-Decoder Adversarial Reconstruction (DEAR) Network for 3D CT from Few-View Data

**DOI:** 10.3390/bioengineering6040111

**Published:** 2019-12-09

**Authors:** Huidong Xie, Hongming Shan, Ge Wang

**Affiliations:** Biomedical Imaging Center, Department of Biomedical Engineering, Center for Biotechnology & Interdisciplinary Studies, Rensselaer Polytechnic Institute, 110 Eighth Street, Troy, NY 12180, USA; xieh2@rpi.edu (H.X.); shanh@rpi.edu (H.S.)

**Keywords:** deep encoder-decoder adversarial network (DEAR), generative adversarial network (GAN), few-view CT, sparse-view CT, machine learning, deep learning

## Abstract

X-ray computed tomography (CT) is widely used in clinical practice. The involved ionizing X-ray radiation, however, could increase cancer risk. Hence, the reduction of the radiation dose has been an important topic in recent years. Few-view CT image reconstruction is one of the main ways to minimize radiation dose and potentially allow a stationary CT architecture. In this paper, we propose a deep encoder-decoder adversarial reconstruction (DEAR) network for 3D CT image reconstruction from few-view data. Since the artifacts caused by few-view reconstruction appear in 3D instead of 2D geometry, a 3D deep network has a great potential for improving the image quality in a data driven fashion. More specifically, our proposed DEAR-3D network aims at reconstructing 3D volume directly from clinical 3D spiral cone-beam image data. DEAR is validated on a publicly available abdominal CT dataset prepared and authorized by Mayo Clinic. Compared with other 2D deep learning methods, the proposed DEAR-3D network can utilize 3D information to produce promising reconstruction results.

## 1. Introduction

X-ray computed tomography (CT) is one of the most essential imaging modalities widely used in clinical practices [1]. Even though CT brings overwhelming healthcare benefits to patients, it could potentially increase the patients’ cancer risk due to the involved ionizing radiation. The data from the National Lung Screening Trial indicate that annual lung cancer screening with low dose CT could significantly reduce lung cancer related mortality [2]. If the effective dose of a routine CT examination is reduced to less than 1 mSv, the long term risk of CT scanning can be negligible. In the past years, numerous deep learning based CT denoising methods were proposed to reduce radiation dose with excellent results [3,4,5]. In parallel, few-view CT is also being actively investigated to reduce the radiation dose, especially for breast CT [6] and C-arm CT [7].

Few-view CT is a challenging problem. Due to the requirement imposed by the Nyquist sampling theorem [8], reconstructing high quality CT images from under-sampled projection data was previously considered an unsolvable problem. With sufficient projection data, analytical methods such as filtered back-projection (FBP) [9] can be used for accurate image reconstruction. However, FBP will introduce severe streak artifacts when projection data are limited. Numerous iterative reconstruction algorithms were proposed to incorporate prior knowledge for suppressing image artifacts in few-view scans. Well known methods include the algebraic reconstruction technique (ART) [10], the simultaneous algebraic reconstruction technique (SART) [11], expectation maximization (EM) [12], etc. Even though these iterative methods do improve image quality, they are usually time consuming and still not able to produce clinically acceptable results in many cases. In the context of this article, clinically acceptable images are reconstructed few-view images that can be used for diagnosis with minimal streak artifacts and clearly shown lesions/subtle details. However, traditional image quality metrics have been used in our paper to quantify image quality. Recently, with the assistance of the graphics processing unit (GPU) and big data, deep learning has become a new frontier of tomographic imaging and gives new opportunities for few-view CT reconstruction [13,14].

Deep learning has been now well recognized in the field of medical tomographic imaging [15]. Several methods were proposed to resolve few-view CT issues in a data driven fashion. For example, based on convolutional neural network (CNN) [16], Jin et al. [17] proposed a FBPConvNet algorithm to remove streak artifacts in the 2D image domain, which utilized FBP measurements as input. Lee et al. used a similar CNN structure to eliminate artifacts in the sinogram domain [18]. Chen et al. designed a Learned Experts’ Assessment-Based Reconstruction Network (LEARN) [19] to map sparse sinogram data directly to a tomographic image, which combines a CNN [16] and a classic iterative process under a data driven regularization. Inspired by the FBP workflow, Li et al. published their iCT-NET [20], which is a deep learning method with a common CNN architecture, to perform CT reconstruction in various special cases and consistently obtain decent results. Our recently published Dual Network Architecture (DNA) network [21] addressed the few-view CT issue by learning a network based reconstruction algorithm from sinogram data. However, none of these proposed methods were designed to perform 3D image reconstruction, subject to potential loss in 3D context.

There are a few publications in the literature trying to use 3D convolutional layers to preserve 3D information during image reconstructions. Low-Dose X-ray Tomography with Generative Adversarial Networks (TomoGAN) [22], proposed by Liu et al., is a novel deep learning based method for low dose CT denoising and few-view CT de-striping. On the other hand, our previous work, Contracting Path-based Convolutional Encoder-decoder (CPCE) network [2], transfers a 2D trained network to a 3D counterpart. Both ways take 3D image patches as input and use 3D convolutional layers to extract 3D feature maps. However, both methods aim at generating a single output image from multiple input slices. Put differently, their methods are a hybrid network consisting of 2D and 3D convolutions, which is also called 2.5D in some papers. The innovative aspect of our paper lies in that, instead of generating a signal 2D output image from multiple adjacent slices, the proposed DEAR aims at generating 3D images directly from few-view 3D input. Since multiple image slices are generated at once through reconstruction during both training and testing phases, our proposed method not only takes advantage of 3D convolutional layers to extract spatial information, but also allows a higher computational efficiency than the TomoGAN and CPCE.

In this paper, we propose a deep encoder-decoder adversarial reconstruction network (DEAR) for 3D CT from few-view data, featuring a direct mapping from a 3D input dataset to a 3D image volume. In diagnosis, radiologists need to extract 3D spatial information by looping adjacent slices and form contextual clues. Therefore, it is reasonable and even necessary to use 3D convolutional layers for maximally avoiding streak artifacts in a batch of adjacent reconstructed image slices. The main contributions of our DEAR-3D network are summarized as follows:

(1) DEAR-3D utilizes 3D convolutional layers to extract 3D information from multiple adjacent slices in a generative adversarial network (GAN) [23] framework. Different from reconstructing 2D images from 3D input data [2], DEAR-3D directly reconstructs a 3D volume, with faithful texture and image details; and
(2) An extensive comparative study was performed between DEAR-3D and various 2D counterparts to demonstrate the merits of the proposed 3D network.

The rest of this paper is organized as follows. Section 2 introduces the DEAR-3D model, its 2D counterparts, and the GAN framework utilized in the proposed model. Section 3 describes our experimental design and results, in comparison with other state-of-the-art models for few-view CT. Finally, Section 4 presents discussions and concludes this paper.

## 2. Methodology

3D CT image reconstruction can be expressed as follows:

(1) $$I=R^{-1}\left(S\right)$$

where $I\in R^{N_{s}\times N\times N}$ denotes a 3D image volume reconstructed from sufficient projection data, where *N* and $N_{s}$ denote the width/height of input images and number of images acquired from a particular patient, respectively. $S\in R^{N_{v}\times N_{d}\times N_{r}}$ denotes the corresponding interpolated 3D sinogram from a spiral cone-beam scan. $N_{v}$, $N_{d}$, and $N_{r}$ denote the number of views, the number of detectors per row, and the number of detector rows, respectively, and $R^{-1}$ is the inverse operator to reconstruct the CT image volume, such as a typical cone-beam reconstruction formula or algorithm [24,25,26,27] when sufficient projection data are obtained. However, when the number of data (linear equations) is not sufficient to resolve all the unknown voxels in the few-view CT setting, streak artifacts will be introduced in the reconstructed images, and it becomes highly non-trivial to reconstruct high quality images. Deep learning (DL) promises to extract features in reference to extensive knowledge hidden in big data. With a large amount of training data, task specific and robust prior knowledge can be taken advantage of in establishing a relationship between few-view data/images and the corresponding full-view images. Such a deep network can be formulated in Equation (2),

(2) $$I_{\mathrm{FullV}}=T\left(I_{\mathrm{FewV}}\right)$$

where $I_{\mathrm{FullV}}$ and $I_{\mathrm{FewV}}$ denote a 3D image volume reconstructed from sufficient projection data and the counterpart reconstructed from insufficient projection data, respectively, and *T* denotes our DEAR-3D network to remove artifacts caused by the few-view problem.

### 2.1. Proposed Framework

**Figure 1:**
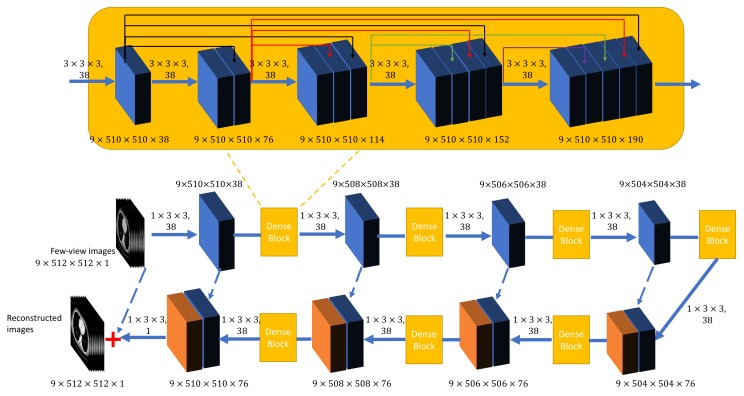
The overall structure of the proposed generator network *G* in the DEAR-3D network. Note that the numbers beside each rectangular block represent the dimensionality of the feature maps, and the dotted blue lines represent conveying paths. The images are the input and output examples for the proposed method. The numbers above each solid blue arrow, separated by a comma, indicate the dimensionality of the convolutional kernel and the corresponding number of filters. Solid black, red, green, and purple arrows represent conveying paths inside dense blocks.

The overall network architecture is shown in Figure 1. The proposed DEAR-3D network is optimized in a Wasserstein generative adversarial network (WGAN) framework [28], which is currently one of the most advanced frameworks. In this study, the proposed framework consists of two components: a generator network *G* and a discriminator network *D*. *G* aims at directly reconstructing a 3D image volume from a batch of 3D few-view image slices. *D* receives images from both *G* and the ground truth dataset, trying to distinguish whether the input is real. Both networks optimize themselves during the training process. If an optimized network *D* can hardly distinguish fake images (from *G*) from real images (from the ground truth dataset), then the generator network *G* fools the discriminator *D* successfully. By design, the introduction of *D* also helps to improve the texture of reconstructed images.

Different from the vanilla generative adversarial network (GAN) [23], WGAN replaces the logarithm term in the loss function with the Wasserstein distance, improving the training stability. In WGAN, the one-Lipschitz function is assumed with weight clipping. However, the work in [29] pointed out that weight clipping may be problematic in WGAN and suggested to replace it with a gradient penalty term, which is used in our proposed method. Hence, the objective function of the GAN framework is expressed as follows:

(3) $$\begin{array}{c} \max_{\theta_{G}}\min_{\theta_{D}}\underset{Wasserstein\;distance}{\underset{︸}{\{E_{I_{\mathrm{FewV}}}\left[D\left(G\left(I_{\mathrm{FewV}}\right)\right)\right]-E_{I_{\mathrm{FullV}}}\left[D\left(I_{\mathrm{FullV}}\right)\right]}}+\lambda \underset{gradient\;penalty}{\underset{︸}{E_{\bar{I}\left(\alpha \right)}[(∥\nabla \left(\bar{I}\left(\alpha \right)\right){∥}_{2}{-1)}^{2}]\}}} \end{array}$$

where $I_{\mathrm{FewV}}$ and $I_{\mathrm{FullV}}$ represent few-view 3D image volume and full-view 3D image volume, respectively, $E_{a}\left[b\right]$ denotes the expectation of *b* as a function of *a*, $\theta_{G}$ and $\theta_{D}$ denote the trainable parameters of the networks *G* and *D*, respectively, and $\bar{I}\left(\alpha \right)=\alpha \cdot I_{\mathrm{FullV}}+\left(1-\alpha \right)\cdot G\left(I_{\mathrm{FewV}}\right)$. α is uniformly sampled from the interval [0,1]. In other words, $\bar{I}$ represents another batch of 3D image slices between fake and real images. Furthermore, $\nabla (\bar{I}\left(\alpha \right))$ denotes the gradient of $\bar{I}$ with respect to $\theta_{D}$. Lastly, λ is a parameter used to balance the Wasserstein distance and the gradient penalty. The networks *G* and *D* are updated in an iterative manner as suggested by [23,28,29].

### 2.2. Generator Network

The input to the generator *G* is a batch of 3D image slices with dimensionality of $N_{b}\times N_{s}\times N\times N$ where $N_{b}$, $N_{s}$, and *N* denote the batch size, number of adjacent input slices, and dimension of each input image slice. Intuitively, $N_{s}$ should be equal to the total number of image slices of a particular patient, and tissues in all the different 2D planes should relate to each other. However, this is not practical due to an extremely large memory cost. Hence, $N_{s}$ is experimentally adjusted to nine. The structure of the generator *G* is inspired by the U-net [30], originally proposed for biological image segmentation. Since then, the U-net has been utilized for various applications in the field of medical imaging. For example, the work in [2,3] used U-net with conveying paths for CT image denoising; the work in [17,18,21] applied U-net for few-view CT; and the work in [31] for compressed sensing MRI. In DEAR, *G* is a revised U-net with conveying paths and built in reference to DenseNet [32]. The generator *G* consists of four 3D convolutional layers for down-sampling and four 3D transpose convolutional layers for up-sampling a batch of 3D image slices. The dimension of the 3D kernel for down-sampling and up-sampling was set as 1×3×3. In the original U-net [30], a stride of two was used in each down-sampling or up-sampling layer to extract features in different dimensions for segmentation. However, for image reconstruction, down-sampling input images severely may result in a compromised performance because convolutional layers may not be able to recover the images from low-dimensional feature maps accurately. Therefore, a stride of one was used in all the convolutional layers of *G*, and zero-padding was not applied in down-sampling and up-sampling layers. A rectified linear unit (ReLU) activation function was used after each 3D convolutional layer.

A dense block was added after each down-sampling and up-sampling layer. Each dense block contained five 3D convolutional layers to extract 3D image features from the input feature maps. Note that zero-padding was used in all 3D convolutional layers to maintain the dimensionality of the input feature maps. Inspired by ResNet [33], shortcuts were applied to connect early and current feature maps, allowing gradients to flow directly to the current layer from the corresponding earlier layer. Different from ResNet, DenseNet further improves the information flow between layers by connecting all the earlier feature maps to the current layer. Consequently, the $l^{\mathrm{th}}$ layer receives all the feature maps from all previous layers, $x_{0}$, $x_{1}$, $x_{2}$, … , $x_{l-1}$, as the input:

(4) $$x_{l}=T_{l}\left(\left[x_{0},x_{1},x_{2},\ldots ,x_{l-1}\right]\right)$$

where $\left[x_{0},x_{1},x_{2},\ldots ,x_{l-1}\right]$ represents the concatenation of all the feature maps produced by the layers 0,1,2,…,l−1, $T_{l}$ denotes the operation performed by the $l^{\mathrm{th}}$ layer, and is defined as a composite function of a 3D convolutional operation and a ReLU activation. The kernel size and stride were set as 3×3×3 and one, respectively for all the 3D convolutional layers in the proposed dense block. Note that the purpose of DEAR-3D is to learn the inverse amplitude of artifacts in the input images, and therefore, input images are directly added to the last convolutional layer as presented in Figure 1.

### 2.3. Discriminator Network

The discriminator network *D* takes input from either *G* or the ground truth dataset, trying to classify whether the input images are real. In DEAR-3D, the discriminator network has six convolutional layers with 64, 64, 128, 128, 256, and 256 filters, followed by two fully connected layers with the numbers of neurons 1024 and one, respectively. The leaky ReLU activation function is used after each layer with a slope of 0.2 in the negative part. 3D convolutional layers with a 3×3×3 kernel dimension and zero-padding were used for all convolutional layers. The stride was set to two for all the layers.

### 2.4. Objective Functions for Generator

**Figure 2:**
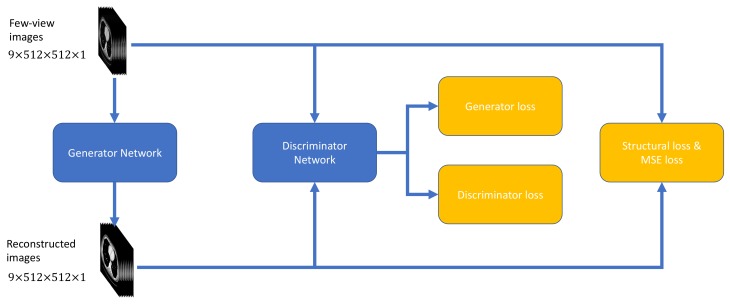
Objective functions used to optimize the proposed DEAR-3D network.

This subsection introduces and evaluates different objective functions used for few-view CT artifact reduction. As shown in Figure 2, a composite objective function was used to optimize DEAR-3D.

#### 2.4.1. MSE Loss

The mean squared error (MSE) [3,34,35] is a popular choice for denoising and artifact removal applications [35]. Nevertheless, it could lead to over-smoothed images [36]. Moreover, MSE is not sensitive to image texture and assumes background noise is white Gaussian noise independent of local image features [37]. The MSE used in the proposed method is expressed as follows:

(5) $$L_{2}=\frac{1}{N_{s}\cdot N\cdot N}\sum_{i=1}^{N_{b}}{∥Y_{i}-X_{i}∥}_{2}^{2}$$

where $N_{b}$, $N_{s}$, and *N* denote the number of batches, the number of input slices, and the image width/height, respectively, and $Y_{i}$ and $X_{i}$ represent the ground truth 3D image volume and 3D image volume reconstructed by *G*, respectively.

#### 2.4.2. Structural Similarity Loss

To overcome the disadvantages of MSE loss and acquire visually superior images, the structural similarity index (SSIM) [37] was introduced in the objective function. SSIM measures structural similarity between two images. The SSIM index is calculated within a convolutional window. The window size was set to 11×11. Compared with traditional pixel-wise measures such as MSE and PSNR, SSIM compares the local structures between the ground truth and reconstructed images. Note that three components were combined to compute SSIM in Equation (6): luminance comparison, contrast comparison, and structure comparison. The SSIM is expressed as follows:

(6) $$SSIM\left(Y,X\right)=\frac{\left(2\mu_{Y}\mu_{X}+C_{1}\right)\left(2\sigma_{YX}+C_{2}\right)}{\left(\mu_{Y}^{2}+\mu_{X}^{2}+C_{1}\right)\left(\sigma_{Y}^{2}+\sigma_{X}^{2}+C_{2}\right)}$$

where $C_{1}={\left(K_{1}\cdot R\right)}^{2}$ and $C_{2}={\left(K_{2}\cdot R\right)}^{2}$ are constants used to stabilize the formula if the denominator is too small, *R* stands for the dynamic range of voxels’ values, $K_{1}=0.01$ and $K_{2}=0.03$, and $\mu_{Y}$, $\mu_{X}$, $\sigma_{Y}^{2}$, $\sigma_{X}^{2}$, and $\sigma_{YX}$ are the means of *Y* and *X*, variances of *Y* and *X*, and the covariance between *Y* and *X*, respectively. Since the maximum value of SSIM was one, the structural loss used to optimize DEAR-3D is expressed as follows:

(7) $$L_{sl}=\frac{1}{N_{b}}\sum_{i=1}^{N_{b}}\left[1-SSIM(Y_{i},X_{i})\right].$$

#### 2.4.3. Adversarial Loss

The adversarial used in DEAR-3D is for the generator to produce realistic images that are indistinguishable by the discriminator network. Referring to Equation (3), the adversarial loss is expressed as follows:

(8) $$L_{al}=-E_{I_{FewV}}\left[D\left(G\left(I_{FewV}\right)\right)\right]$$

The overall objective function of *G* is then expressed as follows:

(9) $$L_{G}=\lambda_{al}\cdot L_{al}+\lambda_{sl}\cdot L_{sl}+L_{2}$$

where $\lambda_{al}$ and $\lambda_{sl}$ are hyper-parameters to balance different loss functions.

### 2.5. Corresponding 2D Networks for Comparisons

To evaluate the performance of the proposed 3D network, a 2D network was built for bench-marking, which is denoted as DEAR-2D. DEAR-2D uses the exact same structure as the DEAR-3D, except that all the 3D convolutional layers in the dense blocks are replaced with 2D convolutional layers. Please note that the number of parameters of DEAR-2D will be less than that of DEAR-3D due to the fact that the dimension of input 2D batches is significantly smaller than the dimension of 3D batches. For a fair comparison, another 2D network was built with an accordingly increased number of training parameters, denoted as DEAR-2D-i. The number of training parameters was increased by increasing number of filters in 2D convolutional layers. Different from DEAR-3D, the 2D counterparts only utilize 2D convolutional layers to extract 2D feature maps from a batch of 2D input images. Therefore, the 2D counterparts aim at reconstructing 2D images instead of 3D images, which may lead to a potential loss in contextual information. Consequently, in DEAR-2D, all the 2D convolutional layers in both the encoder-decoder part and the dense blocks contained 38 filters with a kernel dimension 3×3. On the other hand, in DEAR-2D-i, all the 2D convolutional layers in both the encoder-decoder part and the dense blocks contained 48 filters with kernel dimension 3×3. Table 1 shows the numbers of parameters of the three networks.

Moreover, to demonstrate the effectiveness of different loss functions used to optimize the proposed neural network, 2D and 3D networks with different combinations of loss components were considered for comparison.

## 3. Experimental Design and Results

### 3.1. Dataset and Pre-Processing

A clinical abdominal dataset was used to train and evaluate the performance of the proposed DEAR-3D method. The dataset was prepared and authorized by the Mayo Clinic for “the 2016 NIH-AAPM-Mayo Clinic Low Dose CT Grand Challenge” [38]. The dataset contained a total of 5936 abdominal CT images selected with 1 mm slice thickness. All the images were reconstructed from 2304 projections under 100 peak kilovoltage (kVp), which were used as the ground truth images to train the proposed method. The distance between the X-ray source and the detector array was 1085.6 mm, and the distance between the X-ray source and the iso-center was 595 mm. The pixel size was 0.664 mm. All the images were of 512×512. For data-preprocessing, pixel values of patient images were normalized to be between zero and one. During the training process, four patients (a total of 2566 images) were used for training and six patients (a total of 3370 images) for validation and testing. Patches with dimension 64×64 were cropped with a stride of 32 from the whole images for data augmentation, resulting in a total of 502,936 2D training patches. 2D patches were used to train the DEAR-2D and DEAR-2D-i networks. 3D patches were extracted from the pre-processed 2D patches to train the DEAR-3D network. 3D patches were extracted with a stride of one in the $N_{s}$ dimension. Then, the optimized networks were applicable to images with any image dimension since the proposed DEAR-3D network contained only convolutional layers. The fan-beam Radon transform and fan-beam inverse Radon transform [39] were used to simulate 75-view few-view images. 75-view sinograms were synthesized from angles equally distributed over a full scan range.

### 3.2. Hyperparameter Selection and Network Comparison

In the experiments, all code was implemented in the TensorFlow framework [40] on an NVIDIA Titan RTX GPU. The Adam optimization method was implemented to optimize the training parameters [41] with $\beta_{1}=0.9$ and $\beta_{2}=0.999$. During the training process, a mini-batch size of 10 was selected, resulting in an input with dimensionality of 10×9×64×64×1. The hyperparameter λ used to balance the Wasserstein distance and the gradient penalty was set as 10, as suggested in [29]. The learning rate was initialized as $1\times 10^{-4}$ and decreased by a factor of two after each epoch. The hyperparameters $\lambda_{al}$ and $\lambda_{sl}$ were adjusted using the following steps. First, the proposed network was optimized using only the MSE loss. The testing results were treated as the baseline for fine-tuning the other two hyper-parameters. Then, the SSIM loss was added as part of the objective function. Finally, the adversarial loss was added, and the hyperparameter $\lambda_{al}$ was fine tuned. Through this process, $\lambda_{sl}$ and $\lambda_{al}$ were set to 0.5 and 0.0025, respectively. Please note that $\lambda_{sl}$ and $\lambda_{al}$ were fine tuned for the best SSIM values in the validation set.

For qualitative comparison, the proposed DEAR-3D network was compared with two deep learning based methods for few-view CT image reconstruction, including the FBPConvNet method (a classic U-net [30] with conveying paths to solve the CT problem [17]) and a CNN based residual network [42] (denoted as residual-CNN in this paper). To the best of our knowledge, the network settings we used were the same as the network settings described in the original publications. The dataset used to train all the networks were the same in this study, without any pre-trained model used. All the patient images were also preprocessed in the same way as described in the original papers. The analytical FBP method was used as a baseline for comparison.

Moreover, to highlight the effectiveness of the proposed objective functions used in the 3D architecture, as shown in Table 2, five different networks with different combinations of objective functions were trained for comparison: (1) the DEAR-2D network with only MSE loss and without WGAN (denoted as DEAR-2D${}_{1}$); (2) DEAR-2D with MSE and SSIM, but without WGAN (denoted as DEAR-2D${}_{2}$); (3) DEAR-2D-i with MSE and SSIM loss and without WGAN (denoted as DEAR-2D-i); (4) DEAR-3D with MSE and SSIM loss, but without WGAN (denoted as DEAR-3D${}_{1}$); (5) a full DEAR-3D network with WGAN (denoted as DEAR-3D). Hyperparameters for all five networks were experimentally adjusted using the steps mentioned above.

### 3.3. Comparison with Other Deep Learning Methods

**Figure 3:**
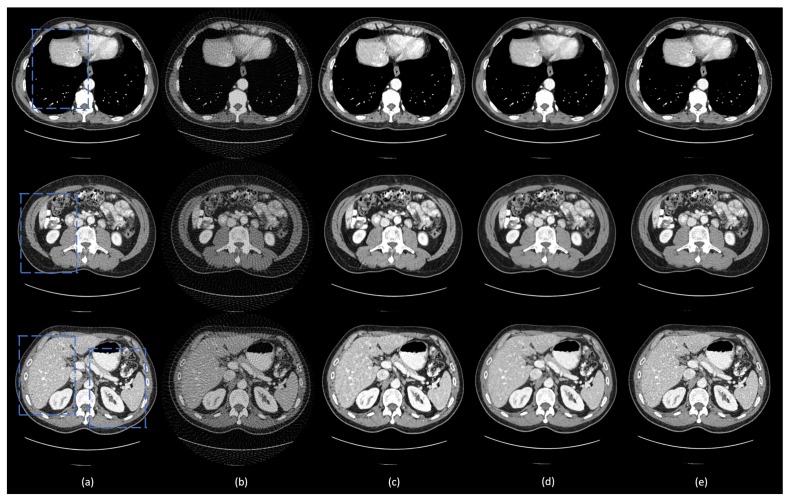
Representative images reconstructed using different methods. (**a**) The ground truth, (**b**) FBP, (**c**) residual-CNN, (**d**) FBPConvNet, and (**e**) DEAR-3D methods. The blue boxes mark the regions of interest (ROIs). The display window is set as [−160, 240] Hounsfield unit (HU) for better visualizing lesions and subtle details.

**Figure 4:**
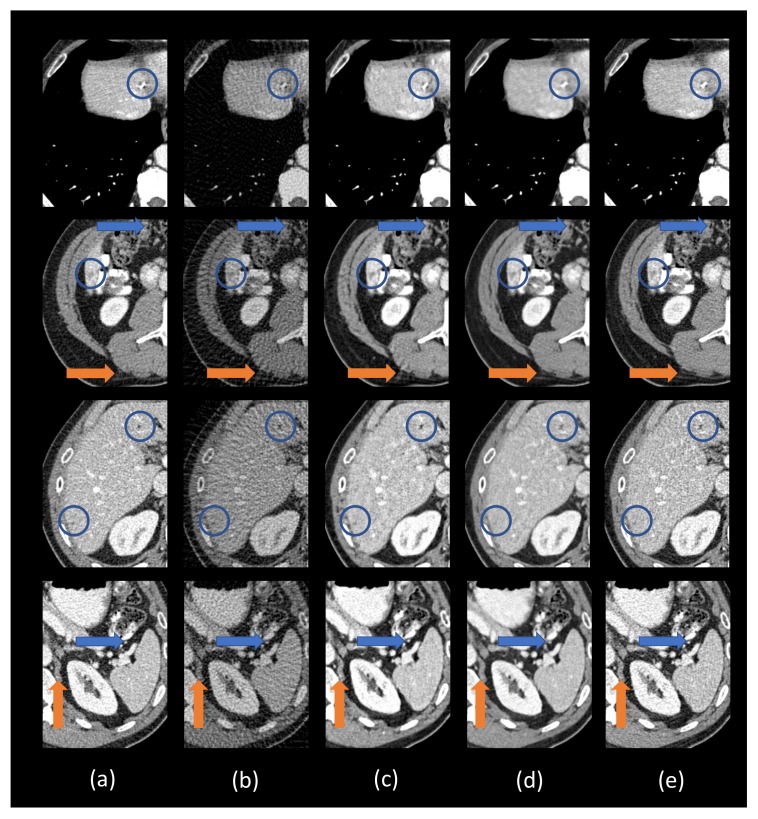
Zoomed-in ROIs (the blue boxes in Figure 3). (**a**) The ground truth, (**b**) FBP, (**c**) residual-CNN, (**d**) FBPConvNet, and (**e**) DEAR-3D. The blue and orange circles mark lesion locations. The blue and orange arrows indicate some subtle details. The display window is [−160, 240] Hounsfield unit (HU).

To visualize the performance of different methods, a few representative slices were selected from the testing set. Figure 3 shows the results using different methods from 75-view few-view images. Three metrics, peak signal-to-noise ratio (PSNR) [43], SSIM, and root mean squared error (RMSE) [44] were computed for quantitative assessment. The quantitative results are shown in Table 3. For better evaluation of the image quality, the regions of interest (ROIs) are marked by rectangles in Figure 3 and are magnified in Figure 4.

The ground truth images and the corresponding few-view images are presented in Figure 3a,b, respectively. As shown in Figure 3b, streak artifacts were clearly visible in the images reconstructed using the FBP method. As shown in the ground truth images in Figure 3a, lesions and subtle details were visible, which were hidden by few-view artifacts in Figure 3a. The results from the 2D based deep learning reconstruction methods (FBPConvNet and residual-CNN) are shown in Figure 3c,d, as well as Figure 4c,d, respectively. These 2D methods can effectively reduce artifacts, but they would potentially miss spatial correlation between adjacent slices, resulting in loss of subtle, but critical details. As shown in the first and second row of Figure 4, FBPConvNet and residual-CNN tended to distort or smooth out some subtle details in the ROIs, but these details were visible in the full dose images reconstructed by the FBP method (indicated by the blue and orange arrows in Figure 4). Moreover, it was observed that residual-CNN was unable to remove streak artifacts in the reconstructed images effectively, especially along the boundaries (the orange and blue arrows in Figure 4). Our proposed method, DEAR-3D, was better at removing artifacts, as well as keeping tiny, but vital details compared to the competitive methods. The proposed DEAR-3D method was also better at recovering image texture than the other methods, and this may be due to the processing capability of the 3D network and the discriminative power of the WGAN framework.

### 3.4. Ablation Analysis

**Figure 5:**
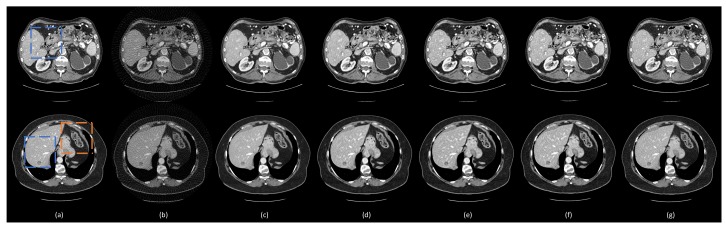
Representative images reconstructed using different methods. (**a**) The ground truth, (**b**) FBP, (**c**) DEAR2D${}_{1}$, (**d**) DEAR-2D${}_{2}$, (**e**) DEAR-2D-i, (**f**) DEAR-3D${}_{1}$, and (**g**) DEAE-3D. The blue boxes mark the regions of interest (ROIs). The orange boxes contain subtle details in the images. The display window is set as [−160, 240] Hounsfield unit (HU) for better visualizing lesions and subtle details.

**Figure 6:**
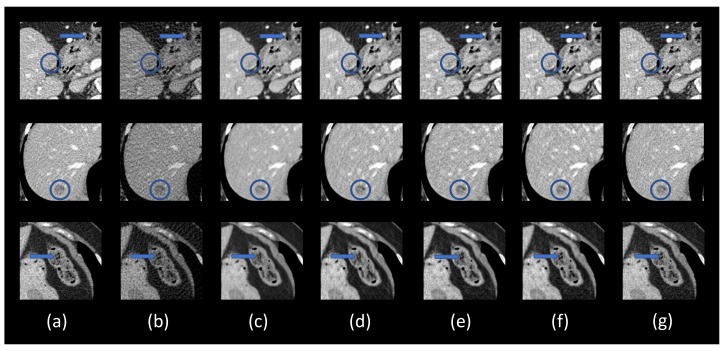
Zoomed-in areas of the lesion (the blue and orange boxes in Figure 5). (**a**) The ground truth, (**b**) FBP, (**c**) DEAR-2D${}_{1}$, (**d**) DEAR-2D${}_{2}$, (**e**) DEAR-2D-i, (**f**) DEAR-3D${}_{1}$, and (**g**) DEAE-3D. The blue circles mark lesion locations. The blue arrows indicate some subtle details. The display window is set as [−160, 240] Hounsfield unit (HU) for better visualizing lesions and subtle details.

This subsection demonstrates the effectiveness of different components in the proposed DEAR-3D network. As mentioned above, five variants of the DEAR-3D network were trained for this purpose. The results are shown in Figure 5, with the corresponding quantitative measurements in Table 4. The zoomed-in regions of interest (ROIs), which are marked by rectangles in Figure 5, are shown in Figure 6. As presented in Figure 6, due to the improper 2D design of the objective function, the DEAR-2D network with only MSE loss tended to smooth out features such as the lesion, leading to an unacceptable image quality (the lesion became barely visible in the first row in Figure 6). Adding SSIM as part of the objective function improved the overall image quality, but due to the lack of 3D spatial context, the 2D based methods were unable to recover subtle details (indicated by the blue arrows in the first and third rows in Figure 6). There was no significant difference observed between the DEAR-2D and the DEAR-2D-i networks. Lastly, the combination of the 3D architecture, WGAN, and the adversarial loss improved image texture and overall image quality, which is desirable in practice. In summary, it was observed that the 2D based methods compromised some details in the reconstructed images (the blue arrows in Figure 6), and by providing information from adjacent slices, the DEAR-3D network performed better than the other methods at removing artifacts and keeping image texture.

## 4. Discussion and Conclusions

Few-view CT may be implemented as a mechanically stationary scanner in the future [45] for health-care and other utilities. Current commercial CT scanners use one or two X-ray sources mounted on a rotating gantry and take hundreds of projections around a patient. The rotating mechanism is not only massive, but also power consuming. Hence, current commercial CT scanners are inaccessible outside hospitals and imaging centers, due to their size, weight, and cost. Designing a stationary gantry with multiple miniature X-ray sources is an interesting approach to resolve this issue [45]. Unfortunately, the current technology does not allow us to assemble hundreds of miniature X-ray sources in a ring for reconstructing a high-quality CT image over an ROI of a decent aperture. Few-view CT is an attractive option. However, streak artifacts would be introduced from a few-view scan due to the insufficiency of projection data. Recently, deep learning has achieved remarkable results for few-view CT, and our proposed DEAR-3D network is a step forward along this direction.

This paper introduced a novel 3D deep encoder-decoder adversarial reconstruction network (DEAR-3D) for directly reconstructing a 3D volume from 3D input data. Compared with 2D based methods [17,18,19,21,42], DEAR-3D avoided the potential loss in the 3D spatial context. Specifically, our proposed network featured: (1) a 3D convolutional encoder-decoder network with conveying-paths; (2) the Wasserstein GAN framework for optimal parameters; and (3) the powerful DenseNet architecture for improved performance.

In conclusion, we presented a novel 3D deep network, DEAR-3D, for solving the few-view CT problem. The proposed method outperformed 3D deep learning methods and promises clinical utilities such as breast cone-beam CT and C-arm cone-beam CT for future research probabilities. In the follow-up investigation, we plan to further improve the network and perform more experiments to optimize and validate the DEAR-3D network.

## Figures and Tables

**Table 1:** Number of parameters used in different networks.

# Parameters	DEAR-3D	DEAR-2D	DEAR-2D-i
	5,123,617	3,459,749	5,519,329

**Table 2:** Summary of deep learning based network architecture and the optimization objective functions for few-view de-artifact methods. The abbreviations for objection functions MSE, SSIM, and AL in this table mean the mean squared error, structural similarity index, and adversarial loss respectively.

	MSE	SSIM	AL
DEAR-2D${}_{1}$	√		
DEAR-2D${}_{2}$	√	√	
DEAR-2D-i	√	√	
DEAR-3D${}_{1}$	√	√	
DEAR-3D	√	√	√

**Table 3:** Quantitative measurements on different methods (mean ± STD). For each metric, the best result is marked as bold. The measurements were obtained by averaging the values in the testing set. The filtered back-projection algorithm is denoted as FBP in this table.

	FBP	FBPConvNet	Residual-CNN	DEAR-3D
PSNR	25.238±0.967	31.437±1.366	30.412±1.269	32.418±1.393
SSIM	0.550±0.031	0.871±0.034	0.870±0.035	0.878±0.033
RMSE	0.055±0.006	0.027±0.004	0.030±0.004	0.025±0.005

**Table 4:** Quantitative measurements for different methods (mean ± STD). For each metric, the best result is marked as bold. Measurements were obtained by averaging the values in the testing dataset. (b) FBP, (c) DEAR-2D${}_{1}$, (d) DEAR-2D${}_{2}$, (e) DEAR-2D-i, (f) DEAR-3D${}_{1}$, and (g) DEAR-3D.

	FBP	DEAR-2D${}_{1}$	DEAR-2D${}_{2}$	DEAR-2D-i	DEAR-3D${}_{1}$	DEAR-3D
PSNR	25.238±0.967	31.556±1.378	31.043±1.360	30.938±1.381	31.068±1.355	32.418±1.393
SSIM	0.550±0.031	0.861±0.033	0.868±0.034	0.865±0.033	0.873±0.033	0.878±0.033
RMSE	0.055±0.006	0.027±0.004	0.028±0.004	0.029±0.005	0.027±0.004	0.025±0.005
